# A journey in the world of craniofacial development: From 1968 to the future

**DOI:** 10.1111/joa.14057

**Published:** 2024-05-18

**Authors:** Gillian Morriss‐Kay

**Affiliations:** ^1^ Emeritus Professor of Developmental Anatomy, Department of Physiology, Anatomy and Genetics University of Oxford Oxford UK

**Keywords:** coronal suture, cranial neurulation, FGFR2, neural crest, rat/mouse embryos, retinoic acid

## Abstract

This article is based on my talk at the meeting “3rd Advances in Craniosynostosis: Basic Science to Clinical Practice”, held at University College, London, on 25 August 2023. It describes my contribution, together with that of my research team and external collaborators, to the field of craniofacial development. This began with my PhD research on the effects of excess vitamin A in rat embryos, which led to a study of normal as well as abnormal formation of the cranial neural tube. Many techniques for analysing morphogenetic change became available to me over the years: whole embryo culture, scanning and transmission electron microscopy, cell division analysis, immunohistochemistry and biochemical analysis of the extracellular matrix. The molecular revolution of the 1980s, and key collaborations with international research teams, enabled functional interpretation of some of the earlier morphological observations and required a change of experimental species to the mouse. Interactions between the molecular and experimental analysis of craniofacial morphogenesis in my laboratory with specialists in molecular genetics and clinicians brought my research journey near to my original aim: to contribute to a better understanding of the causes of human congenital anomalies.

## Graphical abstract

Effects of retinoid excess on craniofacial development: (a) day 20 rat fetuses; (b) newborn baby exposed to maternal 13‐cis retinoic acid.

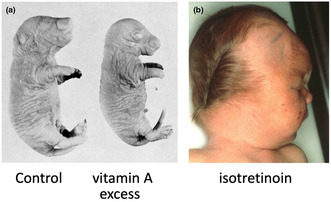

## 1 INTRODUCTION

This article is based on the talk I was invited to give at the meeting “3rd Advances in Craniosynostosis: Basic Science to Clinical Practice”, held at University College, London, on 25 August 2023. It describes some of my research background, from my awakening of interest in craniofacial development, through the subsequent decades, until my retirement from direct involvement in the work. I am grateful to my former collaborators and friends Professors Sachiko Iseki and Andrew Wilkie for inviting me to share my love of this field, and for their practical and intellectual enrichment of my research (summarised in e.g. Morriss‐Kay & Wilkie, 2005).

Because of the personal historical subject matter that forms the substance of this review, the references are mainly to my own work. I hope that my description of the sequence of experiences of my research career may give some encouragement to today's young research workers. The development of new techniques, new collaborative opportunities and personal relationships have as much importance in any research life as they have had in mine. Although the emphasis in this memoir review is on research in which I have been personally involved, its real subject is mammalian craniofacial development. It is a celebration of some of the research in rodent embryos that has contributed to our understanding of normal and abnormal craniofacial development in humans.

The London meeting's subheading “Basic Science to Clinical Practice” describes precisely my aims when I began my PhD study in 1969. I had spent 1968 at Huntingdon Research Centre, a commercial research establishment whose function was to test potential new drugs for safety on behalf of pharmaceutical companies prior to clinical trials – in my section of it, this specifically referred to safety during pregnancy. One of the things I learnt there was that when delivered to pregnant rats, Thalidomide has no effect on the pups, but in rabbits, it causes abnormalities of the limbs equivalent to those caused in humans. This precautionary lesson drew my attention to the importance of selection of the appropriate species when interpreting results using animal models of human disorders and to the significance of mammalian species differences in placental structure and function, which was the major reason for the failure of the human Thalidomide effects to have been picked up in rodents, in which it doesn't cross the placenta. Happily, this caveat doesn't apply to the investigation of genetic disorders inherent in the embryo itself, hence the current importance of the mouse as a model for human genetic diseases.

## 2 AN INTRODUCTION TO VITAMIN A (RETINOL) AND RAT EMBRYO CULTURE

I began my PhD studies in January 1969, supervised by David Woollam in the Cambridge University Anatomy Department, with a brief to investigate the effects of vitamin A excess on prenatal development in rats, which had been reported many years earlier (Cohlan, 1953). The vitamin A preparations were donated by Roche; I was able to apply the techniques I had learnt at Huntingdon to analyse the developmental effects of a single dose of retinyl palmitate on pups of different ages. The craniofacial defects following administration on embryonic day 9 of pregnancy (E9, equivalent to E8 mouse) attracted my curiosity the most: they included abnormal or failed cranial neural tube closure, absent external ears, and severe abnormalities of the jaws and other facial bones (Morriss, 1972, Figures 1a, 2). Analysis of the early developmental defects leading to this outcome revealed that the otocyst was in an abnormally rostral position by E10, level with the mandibular instead of the hyoid arch (Figure 2a,c). The skeletal outcome at E17 included the absence of Meckel's cartilage and an ectopic rod of cartilage in the maxillary region (Figure 2b,d); equivalent abnormalities were present in the bones at E20 (Morriss, 1972). This implied that the skeletal patterning instructions for the facial region had shifted upwards.

**FIGURE 1:**
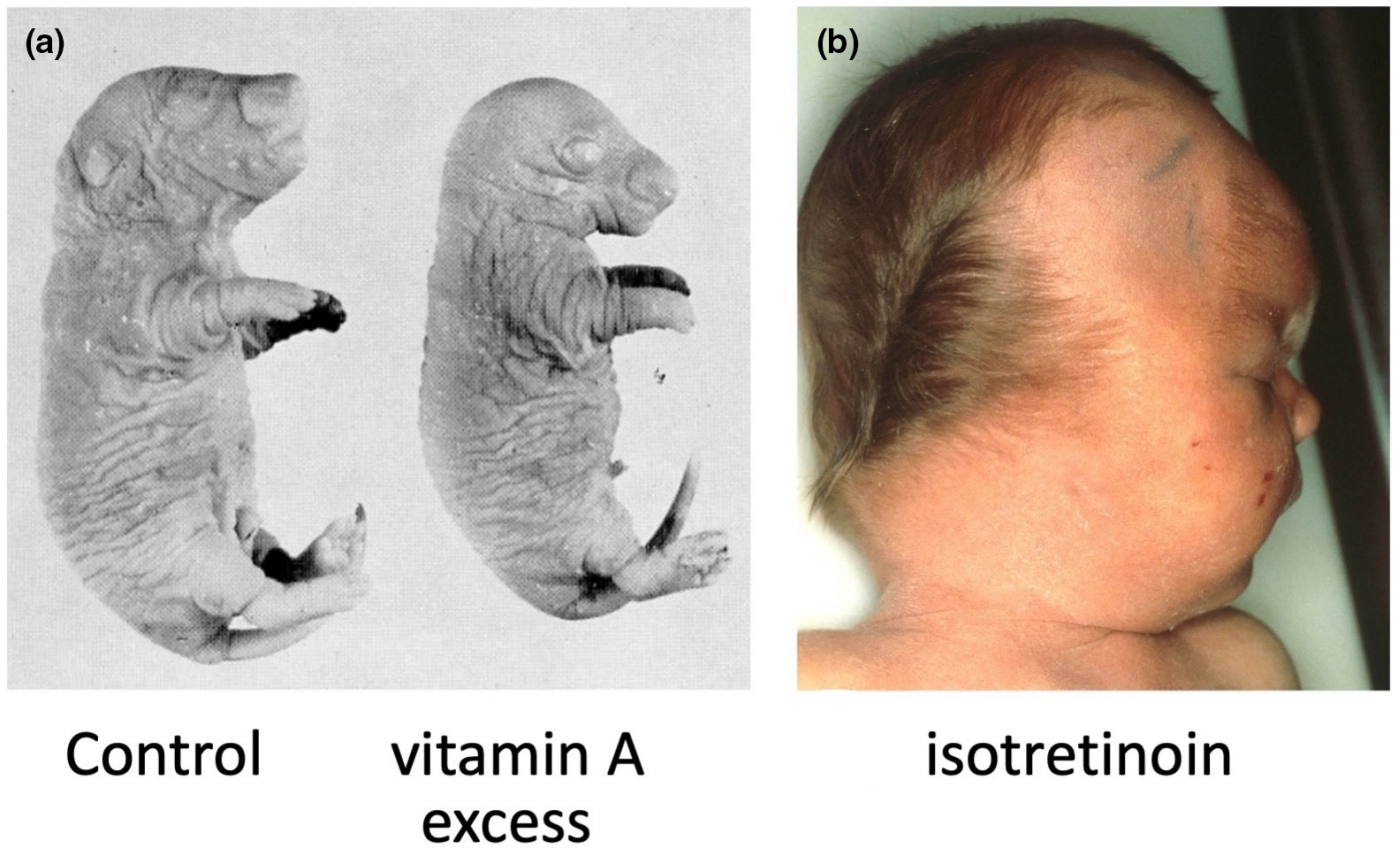
(a) E20 rat fetuses: left, control; right, following maternally administered retinyl palmitate. (b) baby born after exposure to maternal 13‐cis‐retinoic acid (isotretinoin).

**FIGURE 2:**
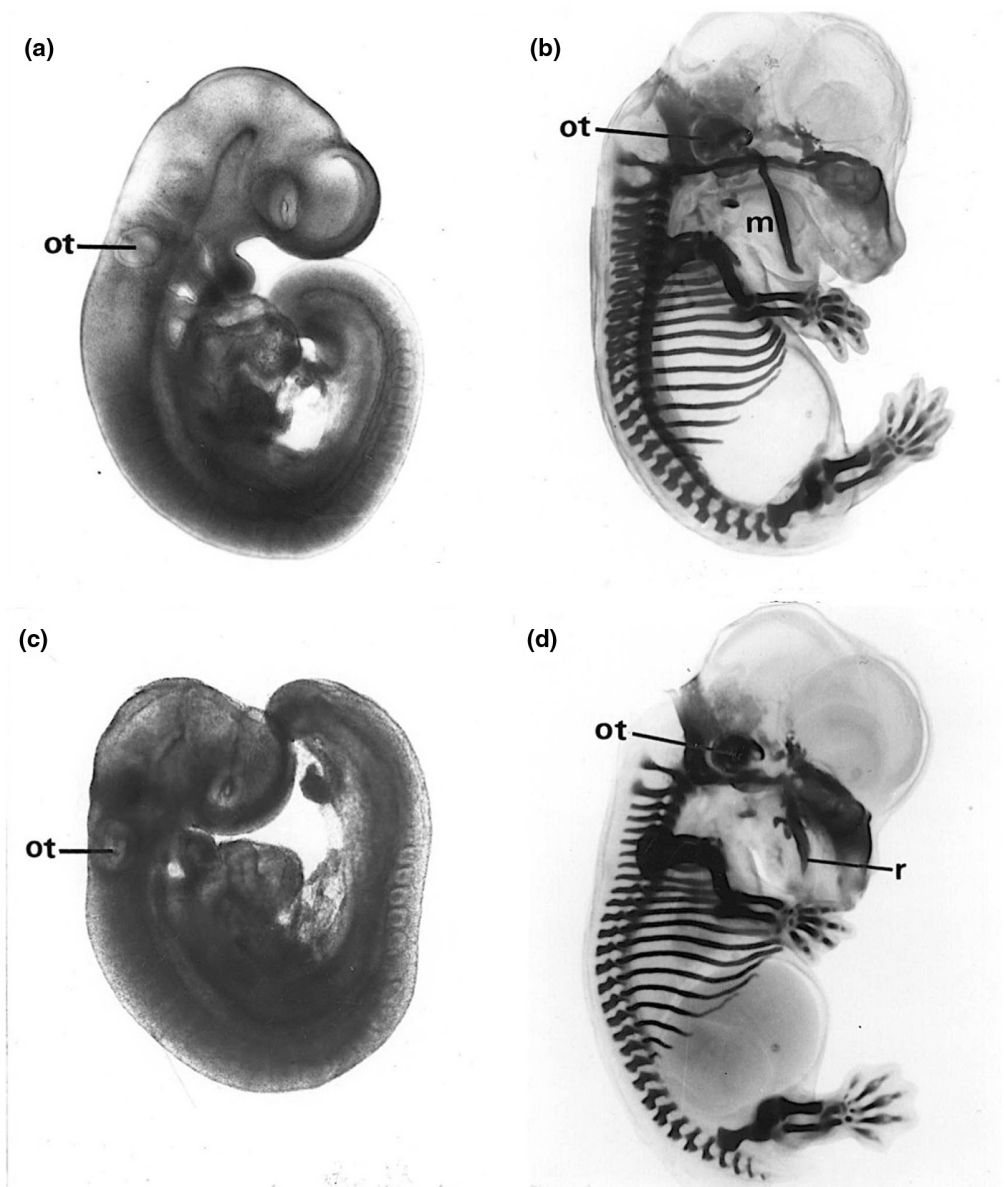
Control (a, b) and maternal retinyl palmitate‐exposed rat embryos (c, d); live embryos at E11 (a, c) and Alcian blue‐stained for cartilage at E17 (b, d), including the otocyst (ot) and Meckel's cartilage (m). In c, the otocyst is positioned level with the first (mandibular) arch instead of the second (hyoid); in d, there is no mandibular (Meckel's) cartilage but a mandible‐like ectopic rod of cartilage (r) is present in the maxillary region.

Some years later, around 1982, Roche marketed a new drug, the retinoic acid isomer 13‐cis retinoic acid (isotretinoin) as an effective treatment for severe cystic acne. It must have gone through the type of “safety in pregnancy” tests that I had carried out at Huntingdon, since it was embargoed for prescription to reproductive age women in the UK. Unfortunately, there was no embargo in the USA, and the drug was also over‐prescribed (Lammer et al., 1985). By 1985, similar effects to those I had described in rat fetuses had been reported in over 200 human babies in the USA (Figure 1b); this number was later reported to be around 1000.

My natural research community in Cambridge was the reproduction group in the Department of Physiology; one of its members was Denis New, who had developed a technique for culturing rat embryos during the early postimplantation period. I was very lucky to have Denis as a colleague, because developmental mammalian research at that time was concentrating on preimplantation development, including in vitro fertilization. I had the extraordinary experience, late one night when I thought I was the only person in the lab, of Bob Edwards running into the room shouting “look, *two* pronuclei” holding a petri dish with a fertilized human egg in it, which he placed under a microscope to show me. This was before the first human in vitro pregnancy. (I subsequently discovered that I wasn't the only person to have had a similar experience!)

Before the development of the chorioallantoic placenta, the nutrition of rodent embryos is via their inside‐out (compared to human) yolk sac, which absorbs nutrients directly from the surrounding maternal blood or from the culture medium. The embryos I learnt to culture developed normally with a 5% or 10% gas phase, but with 20% oxygen (the standard gas phase for culture), neuroepithelial curvature and neural tube closure were delayed. Transmission electron microscopy of cultured and matched ex vivo embryos showed that mitochondrial structure in normally developing embryos during neurulation was characteristic of anaerobic respiration (Morriss & New, 1979); clearly, this is an adaptation to the low oxygen levels associated with yolk sac‐mediated nutrition, both in vivo and in vitro.

The embryo culture technique also enabled me to compare the effects of retinol and retinoic acid on embryonic development, demonstrating that retinoic acid was much more powerful than retinol (Morriss and Steele, 1977). Like maternally administered retinyl palmitate (Figure 2), direct exposure to these two retinoids caused delay or failure of cranial neural tube closure and rostral shift of the otocyst. Interpretation of this result in terms of the molecular pathway involved was not possible until the molecular revolution of the late 1980s brought molecular biology and morphogenesis together. This ground‐shifting change included the discovery of the importance of *Hox* genes in segmental patterning and the role of retinoids in gene expression.

Meanwhile, I needed to move to a different approach to understanding craniofacial development.

## 3 CRANIAL NEURAL TUBE FORMATION: THE ROLE OF EXTRACELLULAR MATRIX

**FIGURE 3:**
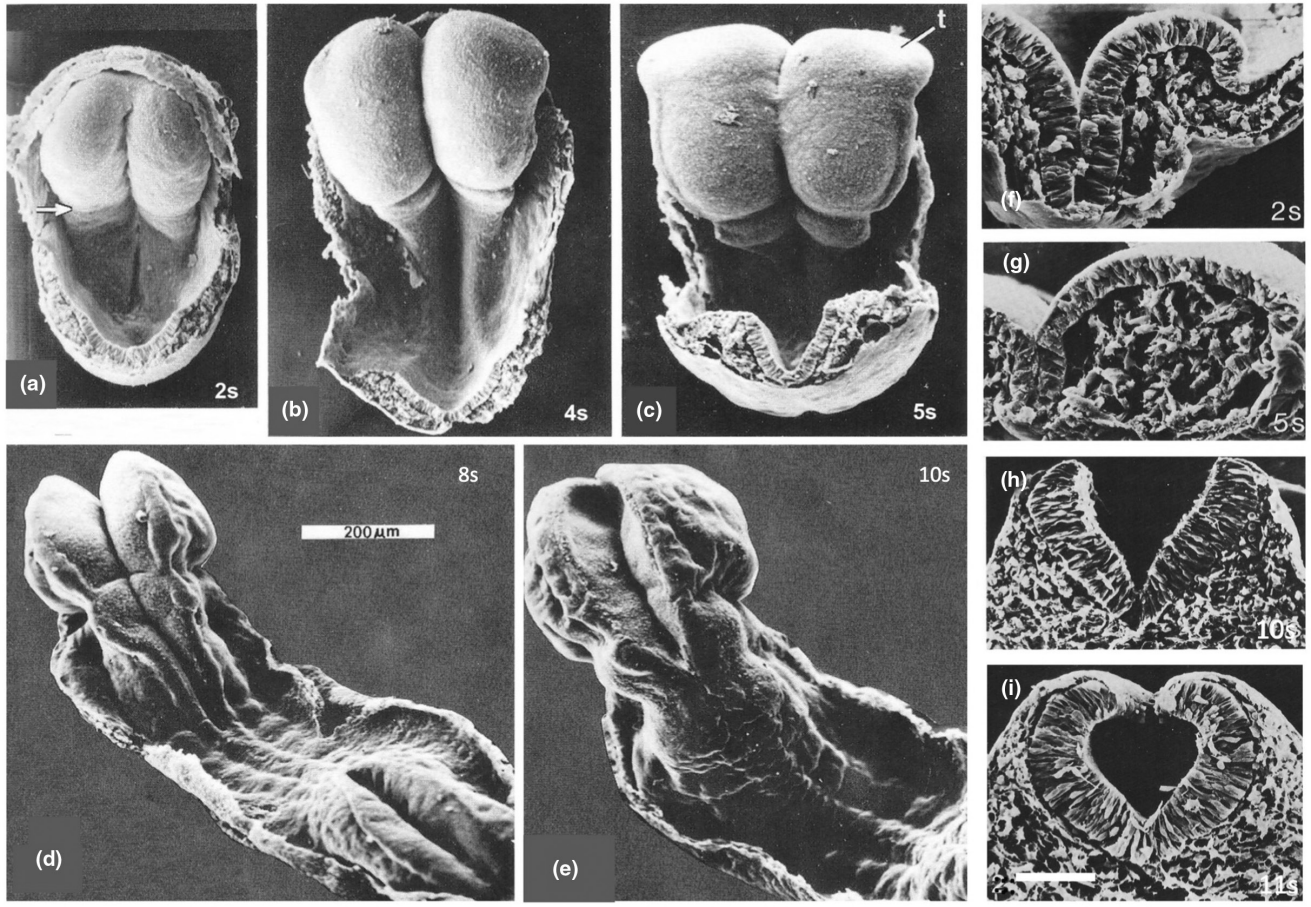
Whole‐mount scanning electron micrographs (SEMs) of rat embryos at 2‐, late‐4‐, 5‐, 8‐ and 10‐somite stages (a‐e). The preotic sulcus (arrowed in a) is a prominent horizontal depressed line across in the upper hindbrain region at all stages shown. At the 5‐somite stage (c), the position of the underlying premigratory neural crest is delineated by a groove parallel to the lateral edges of the midbrain and hindbrain; the telencephalic area (t) of the forebrain has now curved ventrally (away from the plane of view). At the 8‐somite stage (d) the cervical region of the neural tube has closed and the broadest area of open neural folds is V‐shaped; at the 10‐somite stage (e) the open region has a concave surface as the edges approach each other. The preotic sulcus is clearly visible as a transverse groove at all these stages. (f–i) Transverse‐cut SEMs at midbrain/upper hindbrain level at 2‐, 5‐, 10‐ and 11‐somite stages. The neuroepithelial cell number in the transverse plane remains constant at this level as the epithelium thins and broadens; it then becomes pseudostratified (hence thicker) and undergoes concave curvature due to the contraction of transversely oriented apical microfilament bundles. Between the 2‐ and 4‐somite stages, the amount of subepithelial mesenchyme increases in both cell number and extracellular space.

I was very lucky that the Cambridge Anatomy department, unusually at that time, had a scanning electron microscope. This enabled me to begin a detailed study of the morphogenesis of cranial neurulation in whole rat embryos in some detail, alongside transmission electron and light microscopy of both live embryos and sectioned material. Although I didn't complete the SEM observations until I moved to Oxford in 1976, two aspects became clear from the start: (1) unlike cranial neural tube formation in the well‐studied chick embryo, the formation of rat cranial neural folds is at first biconvex; (2) as this shape develops, the amount of subepithelial, widely spaced, mesenchyme increases (Morriss‐Kay, 1981; Figure 3). Later work showed, unsurprisingly, that this pattern of cranial neurulation is essentially identical in mouse embryos.

This observation was in my head when, in 1975, I spent 6 months at the National Institute of Dental and Craniofacial Research, National Institutes of Health, in Bethesda, Maryland, USA. There, I was introduced to the biochemistry of extracellular matrix, specifically proteoglycans and hyaluronic acid (HA). HA is synthesized at the cell membrane; as it extends into the extracellular space, it becomes hydrated and expands its volume up to 1000 times (Whistler & Olsen, 1957). This observation had a clear theoretical link to the increasing amount of extracellular space around the mesenchymal cells underlying the neural folds as they become convex. This link was substantiated by a collaboration with Michael Solursh at NIH and in Iowa, which showed that, when expressed as [^3^H]glucosamine counts per μg protein, there was a 16‐fold increase in the HA level in the embryonic tissue, but not the extraembryonic region, during formation of the primary mesenchyme and neural folds (E7–E10). The sulphated glycosaminoglycans chondroitin sulphate proteoglycan (CS) and heparan sulphate proteoglycan (HS) also increased during the late stage of neurulation, but much less than HA (Solursh & Morriss, 1977).

In addition to histochemical identification of HA, CS and HS in ex vivo embryos, it was possible to investigate their morphogenetic roles by applying specific enzymes to the culture medium, or as injections into the amniotic cavity, and to observe the effect of their loss on cranial neurulation. These experiments used *Streptomyces* hyaluronidase, chondroitinase ABC and heparitinase. HA degradation not only removed the mesenchymal extracellular matrix but decreased the cell cycle time specifically in that tissue, creating smaller embryos that had delayed but complete cranial neural tube closure. When CS was degraded, neurulation was normal until the concave curvature stage, but neural crest cells failed to emigrate. Embryos exposed to heparitinase failed to progress beyond the biconvex stage, indicating that HS is essential for generating the change of neuroepithelial curvature from biconvex to biconcave (Morriss‐Kay, Tuckett and Solursh, 1986; Tuckett and Morriss‐Kay, 1989).

Cranial neurulation had previously mainly been studied in chick and amphibian embryos. The two‐stage process illustrated in Figure 3 is a specifically mammalian feature; it is almost certainly an adaptation to the cranial neural plate being much broader than that of the spinal region and of the cranial region of non‐mammalian vertebrate embryos. I suggest that it would be mechanically impossible for such a broad epithelium to develop a biconcave curvature sufficiently robust to enable the lateral edges to be brought together, even though it is firmly anchored on the midline notochord. The actual morphogenetic process includes a change in epithelial organization from columnar to pseudostratified (Figure 3g, h); this, with the aid of apical microfilament bundles (Morriss & New, 1979) narrows the width of each side, presumably making concave curvature mechanically possible.

## 4 FLUIDITY OF THE NEUROEPITHELIUM AND GROWTH OF THE FOREBRAIN REGION

**FIGURE 4:**
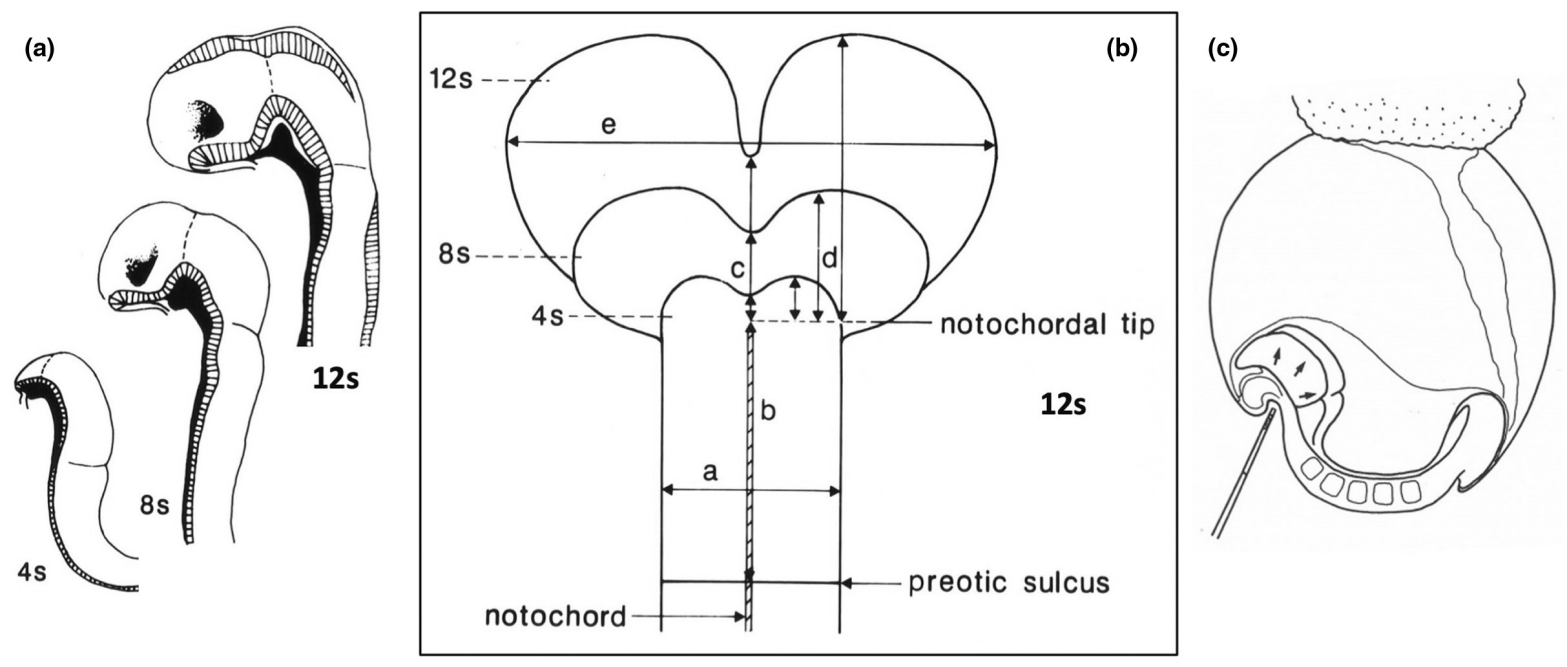
(a) Tracings of some of the bisected SEMs of rat embryos used to assess regional growth during the period of forebrain growth; the notochord is solid black. (b) Cell counts in different planes showed that at the 4‐somite stage, the transverse cell number (a) was 125; this remained constant in the area alongside the notochord, but in the widest part of the expanding area rostral to the tip of the notochord it increased to 260 cells at 8 s and 330 cells at 12 s (e). (c) Injection sites of labelled cells at the 4 s stage prior to culture.

During cranial neurulation, it is clear simply by looking at the growing embryos that the forebrain region is expanding more rapidly than the rest of the cranial neural folds, projecting increasingly further rostral to the tip of the notochord, as seen in tracings of SEM images of bisected embryos (Figure 4a) and in a diagrammatic summary of cell counts (Figure 4b). A study of tritiated thymidine uptake during neurulation of cultured rat embryos was carried out by my research student Fiona Tuckett. Her analysis revealed that during cranial neurulation the number of neuroepithelial cells in the area between the preotic sulcus and the tip of the notochord (midbrain/rostral hindbrain region) is constant; in contrast, the number of cells in the forebrain region is increasing, although the cell division rate is the same in both regions (Tuckett & Morriss‐Kay, 1985).

Another explanation for the differential growth of the forebrain compared to the midbrain and hindbrain was clearly required. It seemed most likely that the neural epithelium acts as a fluid sheet of cells that is flowing rostrally into the expanding forebrain. This was investigated by injecting labelled cells into the convex neural epithelium of 4‐5‐somite‐stage embryos that were then allowed to continue developing in culture (Figure 4c; Morriss‐Kay & Tuckett, 1987). By the end of the culture period the labelled cells had translocated to more rostral positions, confirming the forward movement of these cells within the neuroepithelial sheet bounded by the preotic sulcus caudally and extending into the expanding forebrain region. Furthermore, the orientation of mitotic spindles in the area between the preotic sulcus and the tip of the notochord was predominantly rostrocaudal except at the lateral edges, from which neural crest cells were migrating; in contrast, within the expanding forebrain region rostral to the notochordal tip, their orientation was random. These features are consistent with the observation of a rostrad cell flow, except near the lateral edge. The preotic sulcus, a landmark feature from the 2‐somite stage onwards, appears to be a barrier to the movement of cells in a caudal direction.

## 5 MIGRATION OF MAMMALIAN CRANIAL NEURAL CREST

One of the most interesting aspects of early craniofacial development is the emigration and migration of the cranial neural crest. This had been studied in detail in chick embryos and in chick‐quail chimaeras, especially by Nicole Le Douarin and colleagues in Paris (summarized in the book by Le Douarin & Kalcheim, 1999). Their work had shown that avian cranial neural crest cells populated the pharyngeal arches, but this had not yet been demonstrated in a mammal.

My research student Seong‐Seng Tan first carried out a beautiful scanning electron microscopy study showing cranial crest cells from different cranial levels spreading out from the neuroepithelial edge prior to neural tube closure (Tan & Morriss‐Kay, 1985, Figure 5). He followed this up by transplanting labelled cells into the margin of the neuroepithelium before crest cell emigration and identifying them after 24–48 h in culture in the face and pharyngeal arches (Tan & Morriss‐Kay, 1986). This outstanding work could not have been carried out on mouse embryos, (a) because they develop much more poorly in culture than rats, and (b) their smaller size would have made the already difficult technique of manipulation and injection even more challenging. Seong‐Seng's work left one thing to be done to complete the route map of mammalian cranial neural crest cell migration: confirmation of the pathway from the hindbrain region into the heart. This pathway had been demonstrated in the chick by Kirby et al. (1983); it was established in rat embryos by a Japanese post‐doctoral visitor to my laboratory, Yonetaka Fukiishi, using DiI, a red fluorescent label (Fukiishi & Morriss‐Kay, 1992).

**FIGURE 5:**
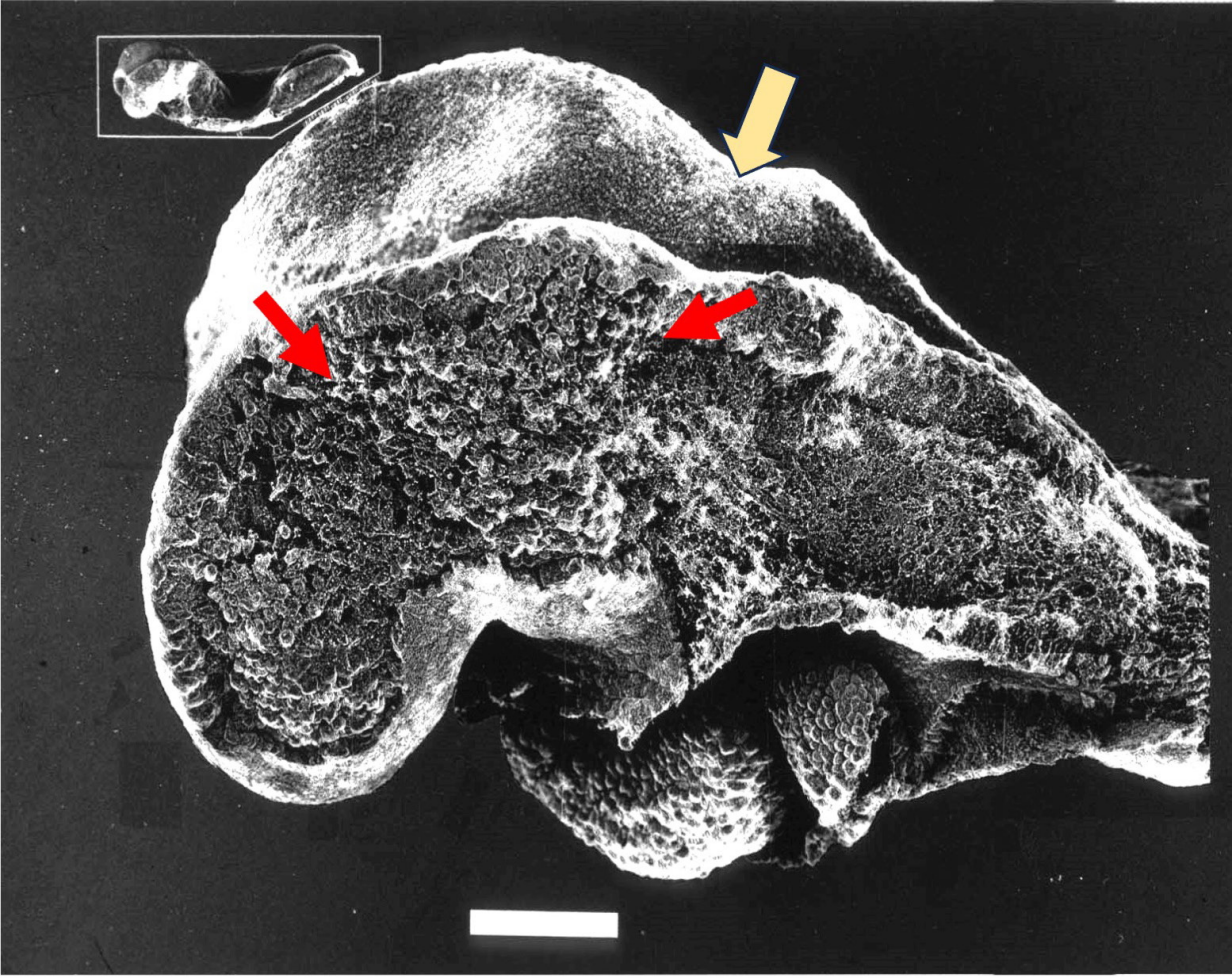
SEM of an 8‐somite stage embryo. The surface ectoderm was removed cleanly with a piece of double‐sided sellotape after drying the specimen. The area between the red arrows is the origin of the frontomaxillary‐mandibular population of NC cells, which can be seen in the streams that have migrated into the forebrain region and into the mandibular (first pharyngeal) arch. The preotic sulcus is indicated by a yellow arrow. The swelling caudal to the preotic sulcus indicates the premigratory hyoid neural crest cell population.

## 6 THE MOLECULAR REVOLUTION: A CHANGE OF APPROACH AND A CHANGE OF SPECIES

The “molecular revolution” of the late 1980s was also a revolution for developmental biologists. It was a magnificent stroke of luck that following the revelatory discoveries in *Drosophila* embryos, the vertebrate species on which the molecular revolution was based was the mouse. Mice have short generation times and are relatively simple to care for. The world of investigative techniques was rapidly expanding, but I had the wrong skill set to profit from it, so I needed collaborations with molecular biologists. What I could offer them was an understanding of mammalian morphogenesis, which they lacked. In addition to the “knock‐out” period of research that analysed the effects of loss of a known gene on embryonic development, there was an important phase of descriptive analysis of developmental gene expression patterns by in situ hybridization of RNA on both sectioned and whole‐mount material. I am grateful to my first research student of this period, Radma Mahmood, for learning the technique of in situ hybridization in an adjacent department and setting it up in my laboratory. She applied it to testing the relationship between Retinoid levels and the expression of development‐related genes including *TGFβ* and *Fgf3* (Mahmood et al., 1992).

**FIGURE 6:**
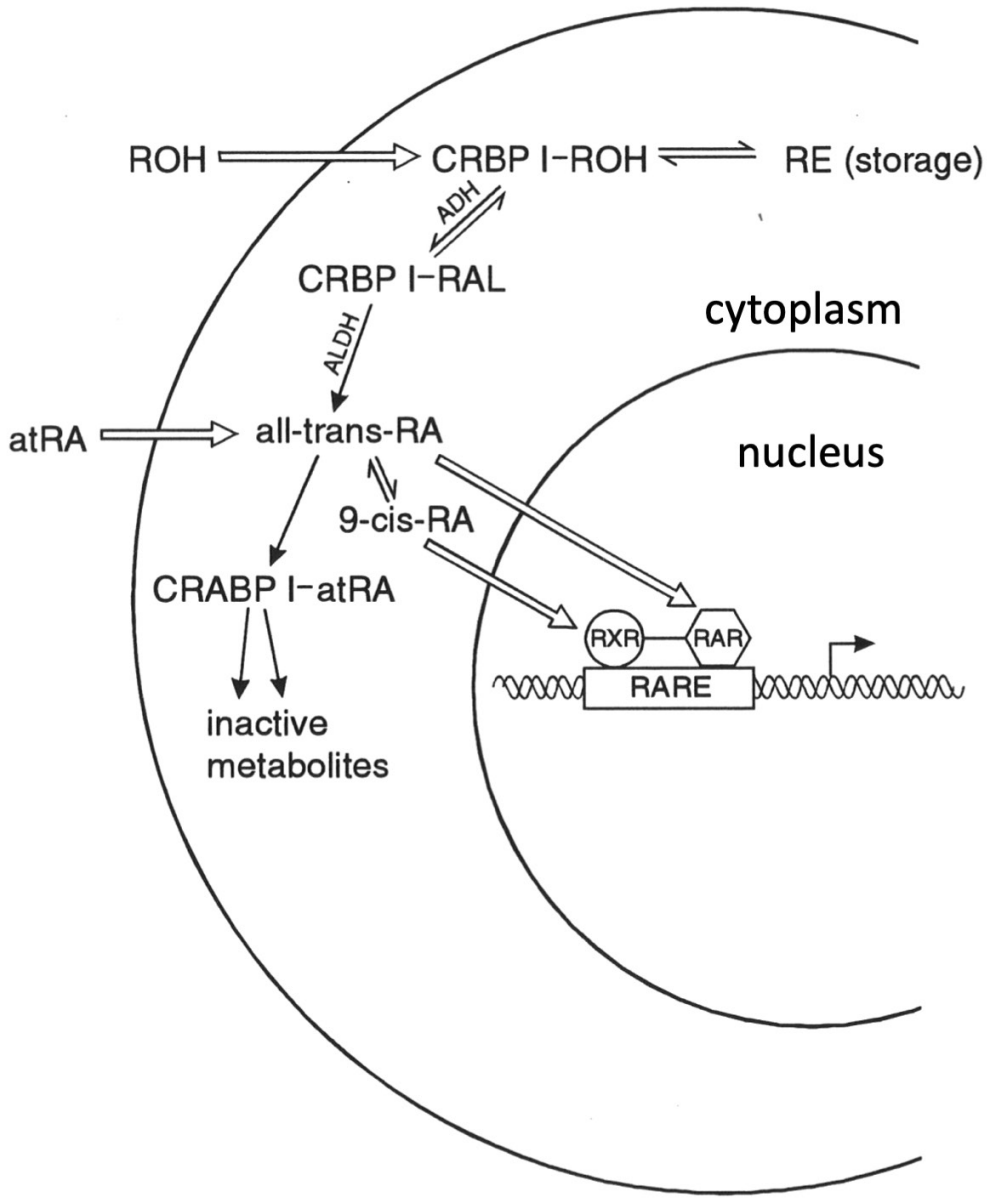
A summary of the findings from the Chambon and Evans groups on the retinoid molecular pathways. atRA, all‐trans retinoic acid; CRBP‐I/CRABP‐I, cellular retinol and retinoic acid binding proteins; RA, retinoic acid; ROH retinol (vitamin A); RAL, retinaldehyde; RARE, retinoic acid response element; RAR/RXR, nuclear response elements for 9‐cis‐RA and all‐trans‐RA; RE, retinyl ester, e.g. retinyl palmitate.

By this time, the world of retinoic acid had been rejuvenated explosively, primarily through the work of Pierre Chambon in Strasbourg and Ron Evans in California (summarized by Chambon, 1996); the new molecular understanding brought about by their research is summarized diagrammatically in Figure 6. In 1989 I was invited by Pierre Chambon to come to his laboratory (Laboratoire de Genetique Moleculaire des Eucaryotes, CNRS) in Strasbourg to help interpret the in situ hybridization patterns of the newly discovered retinoic acid receptors (RAR) and binding proteins (CRABP I and II, and CRBP) during embryonic development. It was a fruitful and enjoyable experience, both intellectually and personally. I co‐authored several publications from this period, beginning with the expression patterns of RARγ (Ruberte et al., 1990). These observations provided a much clearer insight into the mechanisms by which RA acted in the craniofacial region and limbs, as well as elsewhere in the developing embryo.

The effect of retinoid excess on the developing hindbrain could now be revisited. This part of the embryonic brain is segmented into seven rhombomeres (r) with the occipital region (sometimes called rhombomere 8) being unsegmented morphologically (Figure 10) but segmentally influenced by the adjacent somites. In normal vertebrate development, an increasing anteroposterior gradient of RA synthesis is essential for normal hindbrain and spinal cord development (Niederreither et al., 1999; Sakai et al., 2001). The role of each rhombomere in the regional differentiation of r3–r8 depends on the specific pattern of genes of the *Hox‐2* cluster expressed in each of them (Murphy et al., 1989; Wilkinson et al., 1989). In the mouse, there is no *Hox* gene expression in r1 or r2, which are level with the maxillary and mandibular parts of the first pharyngeal arch.

Unlike the other genes of the *Hox‐2* cluster, *Hox*‐2.9 is expressed only in a single rhombomere, r4, flanked by expression of the zinc‐finger gene *Krox‐20* in r3 and r5. Comparison of *Hox‐2.9* expression in normal and RA‐exposed mouse embryos revealed that the rostral shift of the otocyst from its position adjacent to r5/r6 was correlated with the rostral relocation of the *Hox‐2.9* domain from r4 to the r1/2 area of the neuroepithelium (Morriss‐Kay et al., 1991); the r3 domain of *Krox20* was also shifted rostrally and was less well defined. These results are consistent with the interpretation that the rostral shift of the neural crest‐derived facial skeleton is secondary to the RA‐induced effect on the hindbrain Hox gene expression pattern.

## 7 FINALLY, A LINK WITH CLINICAL MOLECULAR GENETICS

When I set out on my life in research, my hope was that I could contribute to understanding the genesis of human craniofacial abnormalities, and possibly even contribute to preventing or ameliorating them. This hope was just pie‐in‐the‐sky at the time, but that was long before the molecular revolution changed the world of developmental genetics. The final phase of my research career took me closer to my original aim than I had expected to achieve, thanks to the two people who introduced me at the Craniosynostosis meeting in August 2023. Andrew Wilkie came into my life in 1994 when he set out to identify the FGF receptor gene responsible for Apert syndrome. Sachiko Iseki arrived in my lab in December of the same year on a 3‐year postdoctoral fellowship from the Human Frontier Science Program. The HFSP administration immediately agreed that she could change from her originally planned project and work on FGF receptor expression and function in the mouse.

**FIGURE 7:**
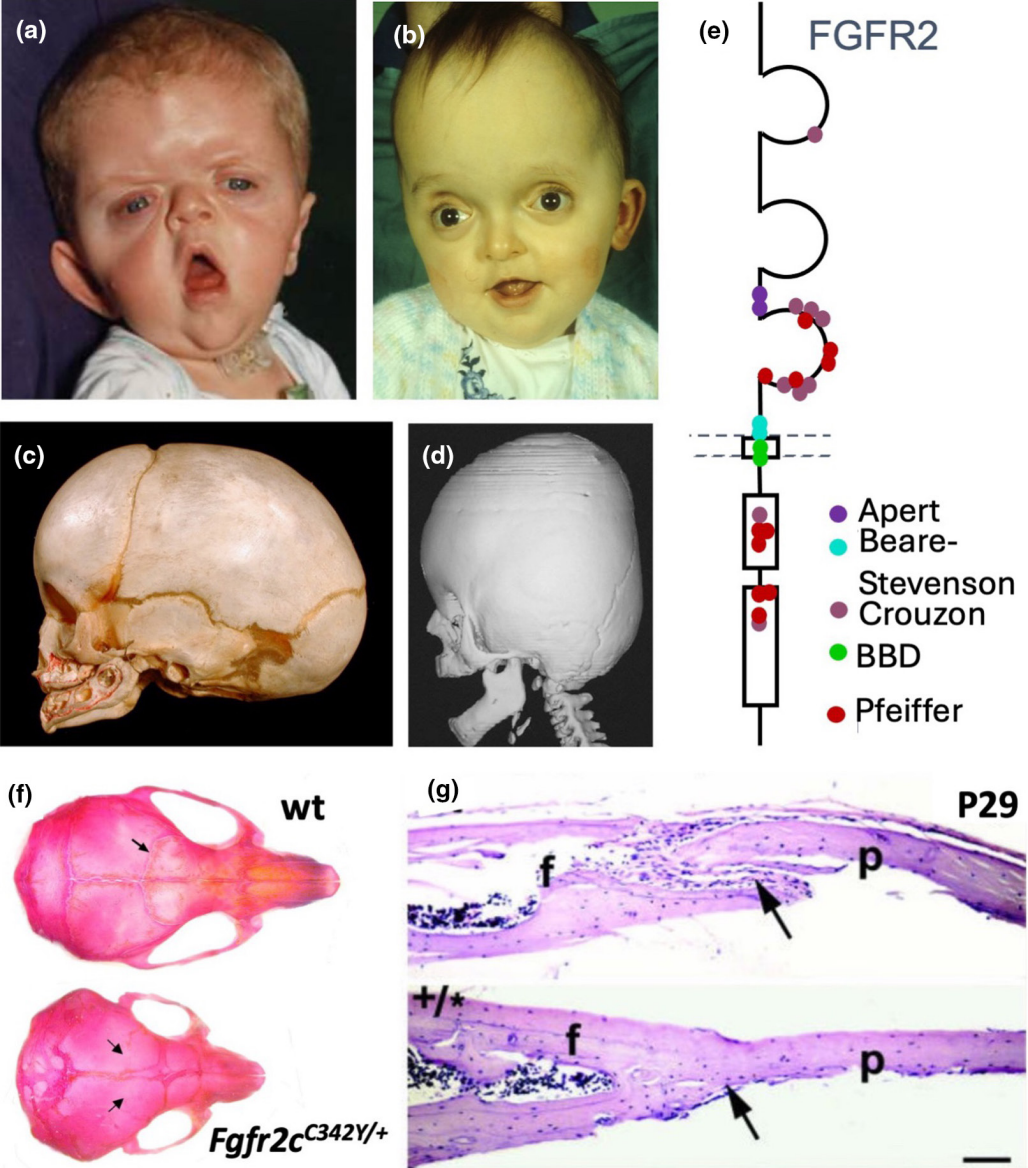
(a) Apert syndrome; (b) Crouzon syndrome; (c) skull of a normal baby; (d) 3D computer tomography scan of a young baby with Crouzon syndrome (courtesy of Philip Anslow). (f) Diagrammatic representation of an FGFR2 monomer showing the positions of craniosynostosis mutations. Dashed lines: position of the cell membrane, outer side above, innerbelow. (e) Diagrammatic summary of FGFR2 with position of craniofacial mutations; dashed lines indicate the cell membrane; outer side top.  (f) P29 wild‐type and *Fgfr2* ^*C342Y/+*^ (Crouzon‐type) mouse skulls; the arrow indicates the coronal suture, which is fused in the mutant skull; the lambdoid suture is also fused. (g) Parasagittal sections through the normal coronal suture of a wild‐type mouse skull and through the fused fronto‐parietal bones of a *Fgfr2* ^*C342Y/+*^ mouse skull (arrowed), both at P29. F, frontal bone; p, parietal bone, BDD, Bent bone dysplasia.

The coincidence of timing couldn't have been better: things were really beginning to shift in understanding the molecular basis of craniosynostosis. Andrew's identification of the Apert mutation in *FGFR2* (Wilkie et al., 1995) immediately followed three related discoveries: *FGFR2* mutations are also responsible for Crouzon, Jackson‐Weiss and Pfeiffer syndromes (Reardon et al., 1994; Jabs et al., 1994 and Muenke et al., 1994; Figure 7). Other human mutations associated with craniosynostosis were found around this time in *MSX2*, *FGFR1*, *FGFR3*, *EFBN1* and *TWIST* genes (see Wilkie, 1997 and Azoury et al., 2017 for references).

FGF receptors are transmembrane receptor tyrosine kinases whose activation depends on dimerization, brought about by the binding of FGF ligands to extracellular immunoglobulin‐like domains, each of which contains a pair of disulphide‐linked cysteines. Craniosynostosis is caused by activating mutations that enable ligand‐independent dimerization. It was a real privilege for me, a biomedical scientist, to attend meetings with the Oxford craniofacial medics at the then Radcliffe Infirmary (now at the John Radcliffe Hospital), to meet the parents and some of the babies with craniosynostosis, and to see how the operations to reopen the closed sutures were carried out. This clinical dimension made a significant contribution to the sense of a wider context to our lab work.

**FIGURE 8:**
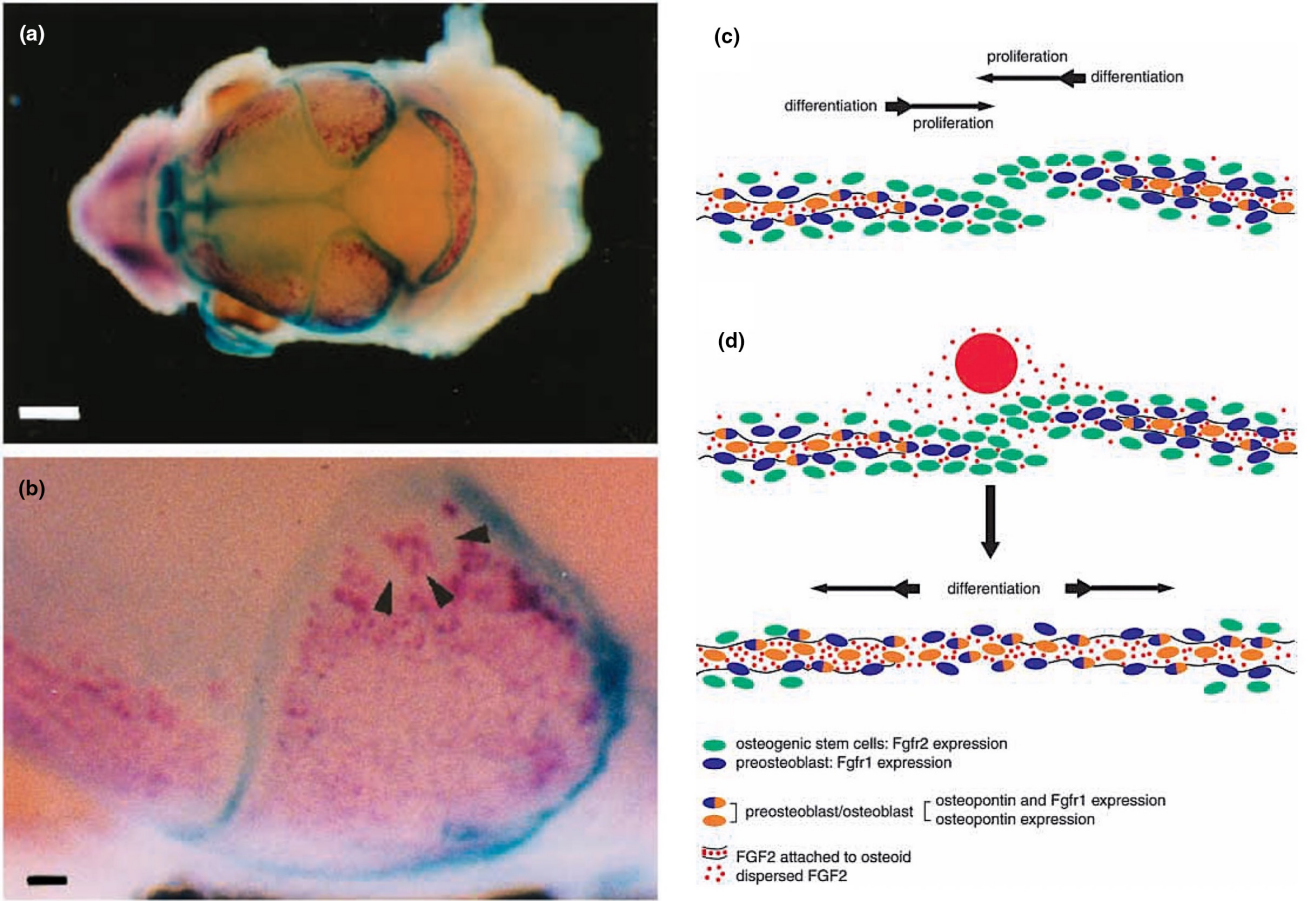
(a, b) Double‐labelled whole‐mount in situ hybridization of *Fgfr2* (blue) and *osteopontin* (magenta) in an E16 mouse skull vault. (c) Interpretation of the proliferative functions of *Fgfr2*‐expressing osteogenic stem cells, the differentiation of *Fgfr1‐*expressing preosteoblasts and their synthesis and secretion of FGF2 ligand. The ligand diffuses away from its source in the area of differentiation so that Fgfr2 molecules are in an environment with a low concentration of ligand, which enables proliferation. (d) Application of an FGF2‐soaked bead to the suture mimics the effects of activating mutation of *Fgfr2*, which is thereby downregulated and *Fgfr1* is upregulated, leading to differentiation. Scale bars: a, 1 mm; b, 100 μm.

Sachiko's first publication from her work in my lab used whole‐mount in situ hybridization to identify the domains of expression of *Fgfr2* and *osteopontin*, a marker for bone differentiation, and used experimental approaches to derive functional information (Iseki et al., 1997). This was important work that not only showed that *Fgfr2* and *osteopontin* expression domains are mutually exclusive but that (a) the *Fgfr2* domain coincides with sites of rapid proliferation and (b) that subcutaneous insertion of a bead soaked in FGF2 to the suture by *ex‐utero* surgery resulted in the downregulation of *Fgfr2* expression and the ectopic expression of *osteopontin* (Figure 8).

**FIGURE 9:**
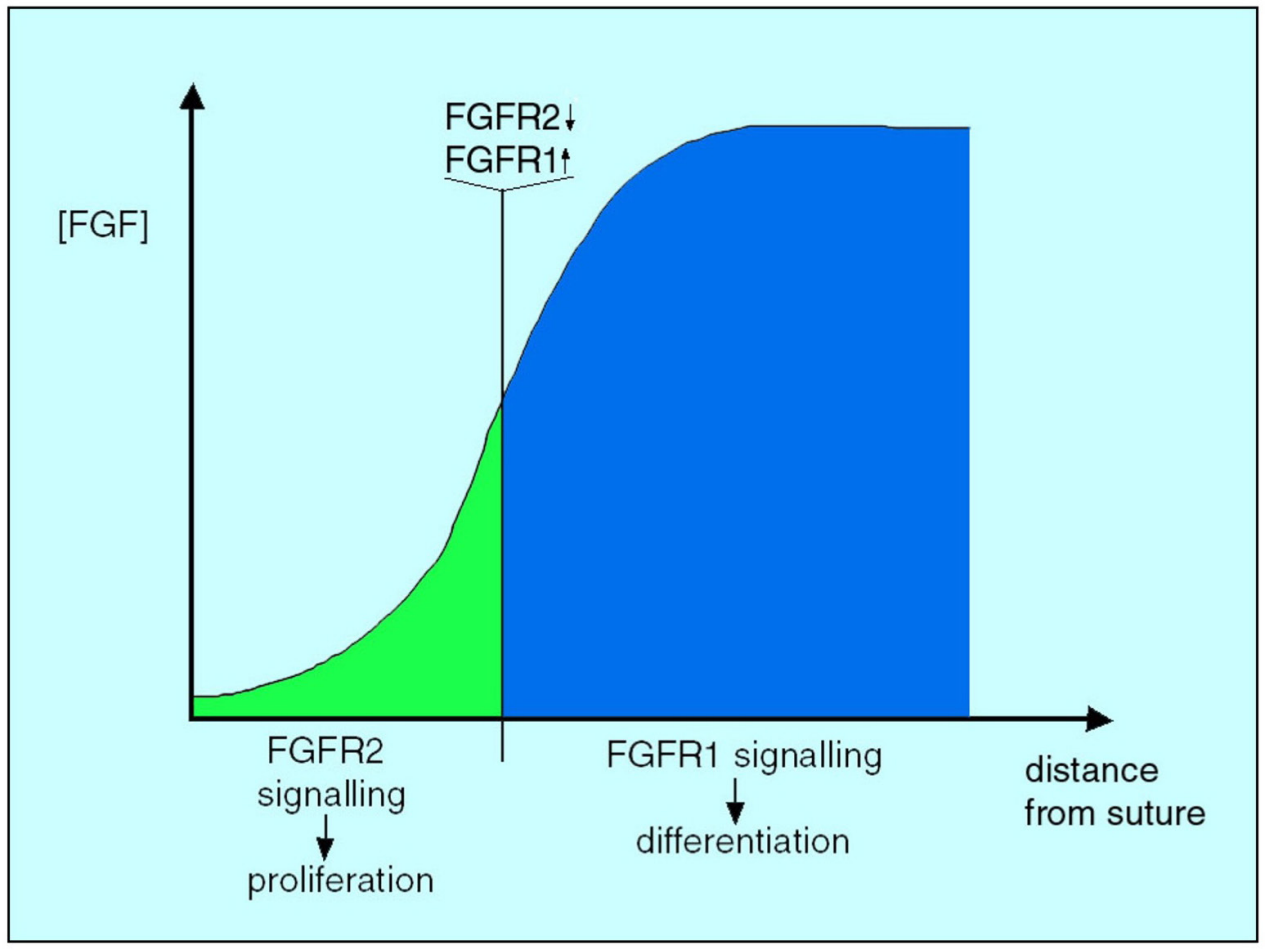
Graphical interpretation of the interdependence of *Fgfr2, Fgfr1* and FGF ligand in growth and differentiation of the coronal suture. The Fgfr2‐mediated proliferation of osteogenic stem cells occurs where FGF concentration is low; as the FGF concentration rises, closer to its source in the *Fgfr1*‐expressing preosteoblasts, *Fgfr2* is downregulated, *Fgfr1* is upregulated and cell behaviour changes from proliferation to differentiation, closing the suture. *From: Morriss‐Kay and Wilkie (2009)*.

This study was followed up with another that demonstrated that while *Fgfr2* has a role in the proliferation of osteogenic stem cells, *Fgfr1* expression is associated with the differentiation of preosteoblasts to osteoblasts (Iseki et al., 1999). This time, FGF2‐bead insertion resulted not only in *Fgfr2* downregulation but also in *Fgfr1* upregulation. These results indicate that in an environment of low‐FGF concentration (i.e. at a distance from its source) FGFR2 signalling results in osteogenic stem cell proliferation. In contrast, where the FGF concentration is higher, FGFR2 is downregulated and signalling through FGFR1 regulates preosteogenic cell differentiation. Hence, the balance between proliferation and differentiation in the coronal suture is controlled by the level of FGF ligand (Figure 9).

A wider point is highlighted by the work on craniosynostosis: progress in the understanding of complex genetic diseases comes from collaborations between clinicians, molecular geneticists and developmental biologists ‐ it is no longer possible to work alone. In addition to the observations on *FGFR2/Fgfr2* described above, these collaborations also confirmed that the developmental expression of *Twist* (Johnson et al., 2000) and *Efnb1* (Twigg et al., 2004) have significant domains that correspond with sites of abnormal development in human coronal synostosis.

The opportunity to work on a specific mouse model for craniosynostosis came from Peter Lonai at the Weissman Institute in Israel. Peter and his colleagues had constructed a mouse with the Crouzon mutation, *Fgfr2c‐C342Y*. The heterozygous *C342Y* mice are near‐perfect mimics of the human Crouzon phenotype, with broad, short skulls and coronal sutures that are completely fused within 4 weeks of birth (Figure 7). I began analysing the embryos and pups during a 2‐week visit to The Weizman Institute before importing some of the mice to Oxford to complete the work (Eswarakumar et al., 2004).

## 8 TISSUE ORIGINS OF THE CORONAL SUTURE

Another major technical breakthrough came in 2000. I was asked to referee two papers that used a recently developed compound‐transgenic mouse that is doubly heterozygous for the *R26R* allele, which encodes β‐galactosidase only in cells and their progeny that express *Cre recombinase* and the *Wnt1‐Cre* transgene, which is specific to neural crest and to some parts of the cranial neural tube. The two papers from Henry Sucov's lab in Los Angeles used this transgenic mouse to analyse the neural crest contributions (a) to the pharyngeal arches and heart (Jiang et al., 2000) and (b) to the mandible and teeth (Chai et al., 2000). I was very excited by this but disappointed that neither paper showed the skull vault. Analysis of the neural crest and mesodermal contributions to the skull vault of avian embryos had already been carried out using the chick‐quail chimaera technique; Couly et al. (1993) interpreted their results as showing that the whole skull vault was neural crest‐derived. Unfortunately, the avian skull has a fused parietal and frontal bone, and the bone they interpreted as the parietal bone in the section that was illustrated was cut through too low and was actually squamosal, which is certainly neural crest‐derived.

**FIGURE 10:**
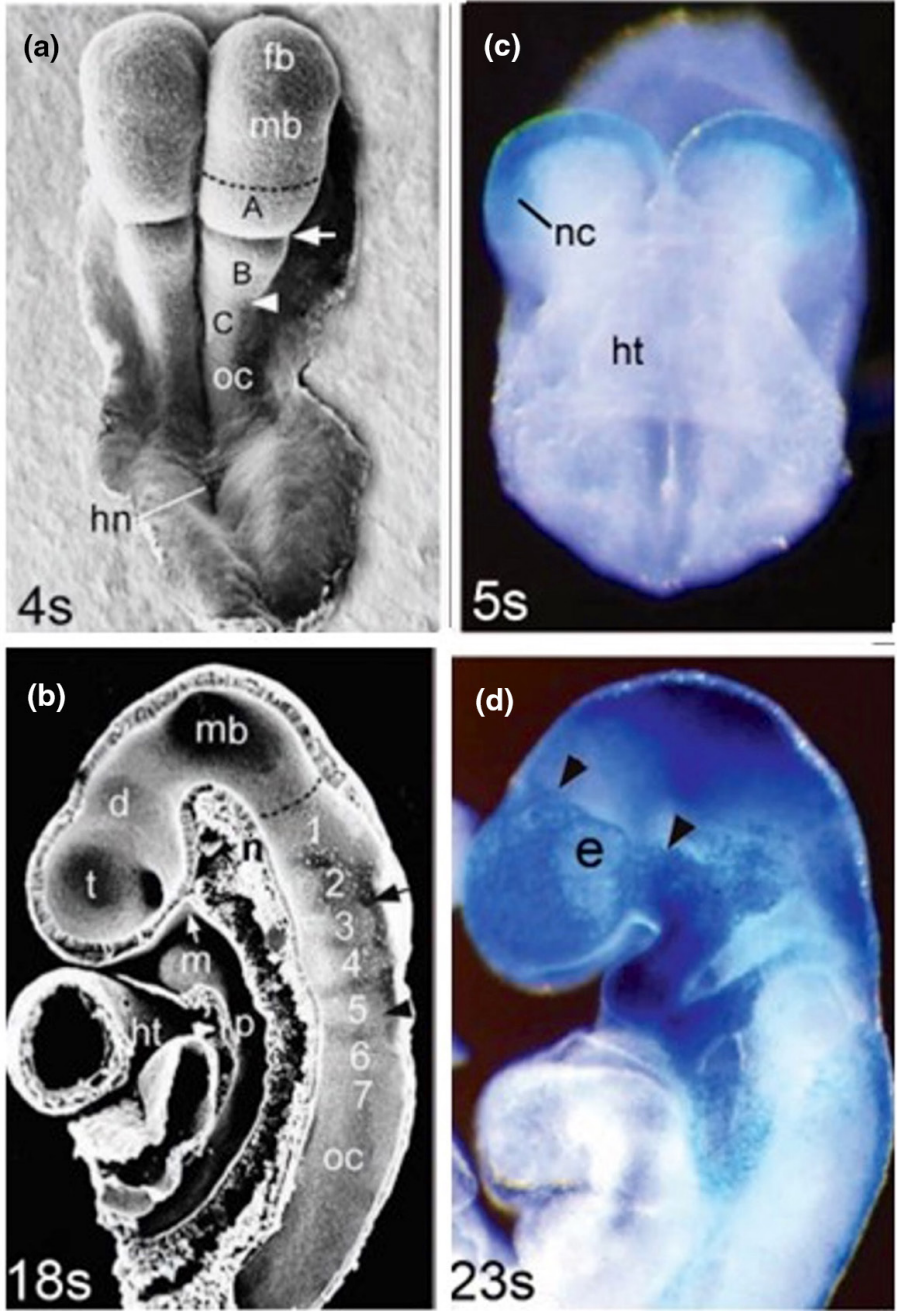
(a) SEM of a 4‐somite‐stage embryo showing the positions of the future forebrain (fb), midbrain (mb), prorhombomeres a, b and c and the occipital region of the hindbrain (oc), the preotic sulcus (arrow), the otic sulcus (arrowhead) and Hensen's node (hn). (b) SEM of a bisected 18‐somite stage embryo showing the internal surface of the brain regions, including the 7 rhombomeres and the unsegmented occipital region (oc), midbrain (mb), diencephalon (d) and telencephalon (t); heart (ht), mandibular arch (m), notochord (n), pharynx (p) and Rathke's pouch (white arrow); black arrows indicate the earlier positions of the preotic and otic sulci. (c) Frontal view of a 5 s *Wnt1‐Cre/R26R* expressing embryo showing an early stage of neural crest (nc) cell migration; the midbrain neural epithelium is also Wnt1‐positive. (d) The 23 s embryo shows the post‐migratory domains of the midbrain/upper hindbrain (r1/2) ‐derived NC cell population around the forebrain, within the maxillary and mandibular regions and in the incipient trigeminal ganglion; the black arrows indicate the clear boundary of the NC domain. The hyoid NC population is present in the second arch. The midbrain and hindbrain neuroepithelium is also *Wnt1‐Cre/R26R* positive.

**FIGURE 11:**
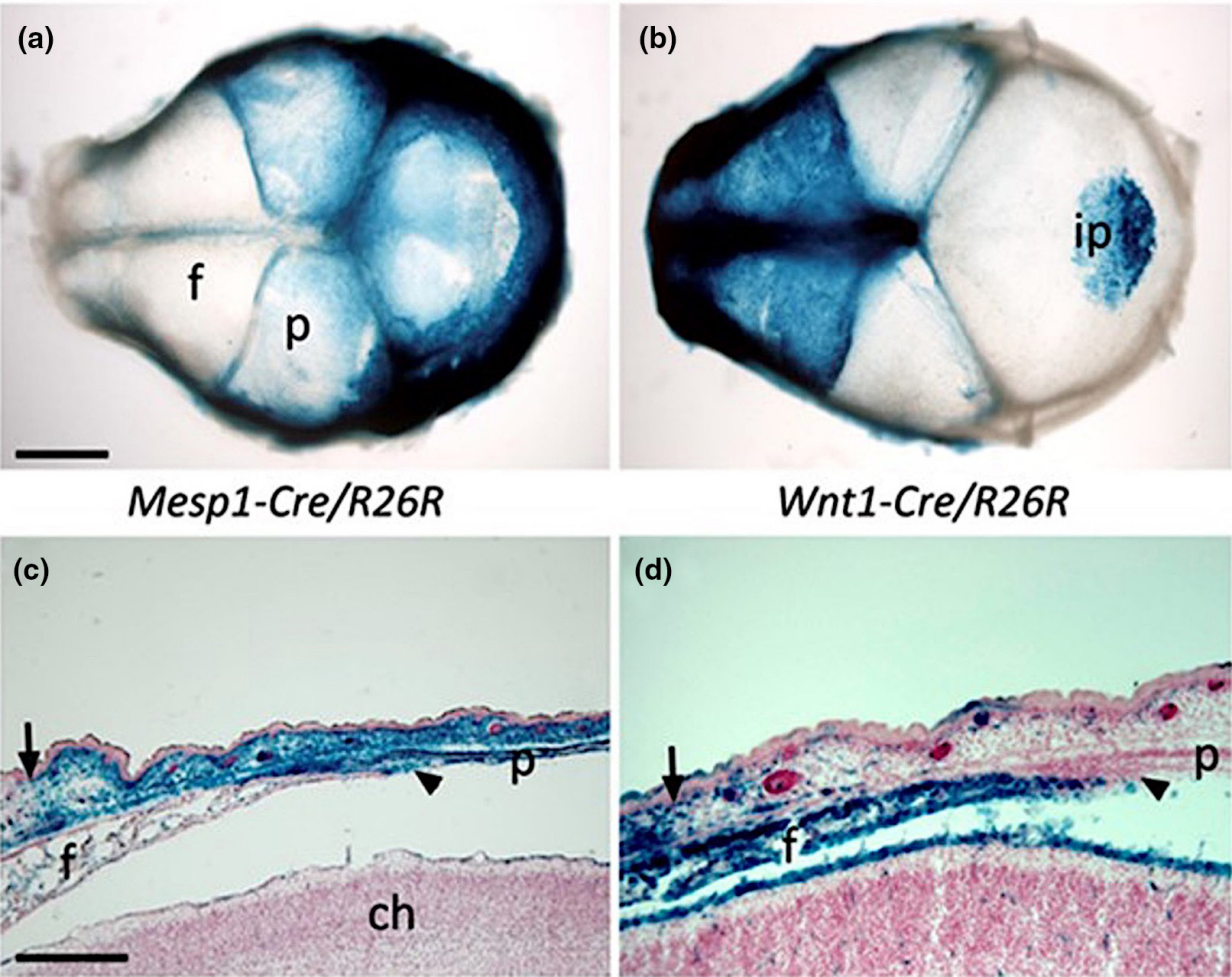
(a, b) E17.5 skull vaults showing mesoderm (*Mesp1‐Cre/R26R*) and neural crest (*Wnt1‐Cre/R26R*) ‐derived regions. (c, d) Horizontal sections of the coronal suture area showing mesodermal and NC origin of tissues in the frontal (f), parietal (p) and interparietal (ip) bones and the meningeal layer over the cerebral hemisphere (ch). The vertical arrow indicates the boundary between mesodermal and NC tissue in the dermis; this boundary has shifted in relation to the position of the coronal suture (arrowhead) due to differential growth of the cranial vault bones and the overlying skin.

The *Wnt1‐Cre/R26R* mouse construct provided a welcome opportunity to elucidate the origins of the skull vault bones in a mammal, and hence, to discover the tissue contributions to the coronal suture. Previously, these could only be assumed by indirect evidence; clarification would shed some light on the evolution of the tissue origins of the bones of the vertebrate skull vault (Morriss‐Kay, 2001). A collaboration with Henry Sucov's group detailed the migration pattern of the cranial neural crest during the period of neurulation (Jiang et al., 2002; Figures 10, 11). To my great delight, it showed that the frontal bone, but not the parietal, is neural crest derived (Figure 11). Other sutures also showed this mesoderm/neural crest tissue juxtaposition – a tongue of neural crest tissue extends from the frontal bone between the two parietal bones, making a tissue sandwich (Figure 11(b)); curiously, there is a patch of neural crest‐derived tissue in the centre of the otherwise mesodermal interparietal (occipital) bone.

The expected criticism came: we hadn't shown that the unlabelled bones were mesoderm. Sachiko solved this problem through collaboration with Yumiko Saga, who had created a double‐transgenic mouse, *Mesp1‐Cre/R26R*, that was a specific tracer for mesoderm (Yoshida et al., 2008). The expression patterns of the two tracers, as predicted, were complementary (Figure 11a–d).

The project of mapping the neural crest and mesodermal contributions to the skull needed one more contribution: analysis of the skull base. This was carried out by a one‐year visitor from Harvard, Brandeis McBratney‐Owen. Brandeis used both the neural crest (*Wnt1‐Cre/R26R*) and mesoderm (*Mesp1‐Cre/R26R*) markers to show that the boundary between them in the cranial base at E16 and P1 is at the tip of the notochord, around the rostral aspect of the hypophyseal foramen (formerly the site of Rathke's pouch, later the pituitary fossa) (McBratney‐Owen et al., 2008).

## 9 CONCLUSIONS

Although I reached the end of my active research career some years ago, I continue to enjoy some intellectual involvement in mammalian embryology through writing, editing and attending seminars. Looking back, I realise how fortunate I have been to have had such a richly enjoyable professional life, and so many valuable interactions with co‐workers. One of the referees of this article asked if I could comment on whether it was more difficult being a female academic in 1968 than it is now. Of course it was, because men then rarely took women seriously or gave them credit for their research. Today we are all just research workers.

Although I have strayed into other parts of the embryo, such as the caudal neuropore and the limbs, understanding more about the development of the skull was my major motivation from the start. Towards the end, this interest became particularly focused on the coronal suture. This is the only cranial suture to have a significant overlap of the adjacent bones, so I was fascinated to observe it in my newborn step‐grandson in 1999: his pale skin and scarcity of hair meant that I could see the sutural overlap, but when he started to cry I could see the two bones slide over each other to widen the suture.

I suggest that in the normal skull, an important function of the coronal suture's overlapping structure is to enable the skull to expand in response to the increased intracranial pressure that crying engenders. This interpretation is consistent with one of my rare clinical experiences, observing an operation to reopen the multiple closed sutures in the skull of a one‐year‐old infant: the poor baby had suffered terrible headaches throughout his short life. I hope that he went on to have a happy life and that current and future advances in clinical approaches to craniosynostosis, aided by advances in biomedical research, will continue to improve the lives of affected children. Specifically, I hope that the integration of biomedical and clinical research will one day enable the behaviour of osteogenic stem cells in synostosed sutures to be regenerated and normalized after surgery.

A final short observation: I find it amusing that hyaluronic acid and retinoic acid, two of the chemicals whose developmental roles I worked on in the 1970s to 1990s, are now so common in the cosmetics industry that it's hard to find a face cream that doesn't contain one or both of them!

## 10 A NOTE ON THE JOURNAL OF ANATOMY

It is a great pleasure to me that this symposium issue is being published in the *Journal of Anatomy*. This was where my first paper was published in 1972, as were a number of others over the years, including reviews in other symposium issues. My long involvement with the Anatomical Society, as a member and for a time as Secretary (1977–2000), led to my appointment in 2002 as Editor of the *Journal of Anatomy*. This came to an end in January 2012, when I wrote my valedictory editorial.

The Anatomical Society, which owns the *Journal*, provides venues at its meetings that have a welcoming, non‐competitive atmosphere and provide the ideal opportunity for research students and post‐docs to present their early‐career work. I am grateful for the many times young members of my group benefitted from this. The Society indulged me with the organization of a symposium on anatomy and art, and the publication of my article on that subject (Morriss‐Kay, 2010). My interest in the history of the *Journal* was written and published as a historical review (Morriss‐Kay, 2016).

## Data Availability

Data sharing is not applicable to this article as no new data were created or analyzed in this study.
